# Mental health among clinicians: what do we know and what can we do?

**DOI:** 10.1007/s00192-021-04805-y

**Published:** 2021-05-03

**Authors:** Mittal Patel, Steven Swift, Alex Digesu

**Affiliations:** 1 St Mary’s Hospital, Imperial College NHS Trust, London, UK; 2 Medical University of South Carolina, Charleston, SC USA

**Keywords:** Mental health, Mental health disorders, Anxiety, Depression, Burnout, Suicide

## Abstract

Mental health and mental health disorders among clinicians remain a taboo, despite increasing evidence showing the direct impact on medical teams and patient care. This editorial is aimed at increasing awareness of mental issues amongst healthcare professionals, identifying perceived barriers to seeking help, and suggesting ways in which to seek help. Mental health disorders, including anxiety and depression, are prevalent from medical school, leading to increased burnout and suicide risks at later stages of a clinician’s career. There is often a reluctance to seek help, particularly amongst the surgical specialties, caused by self-criticism, lack of convenient access and the potential negative impact on medical licensure. This editorial has been written in loving memory of our colleague, friend and board member Dr. Nikolaus Veit-Rubin, who sadly passed away at the beginning of the year. It is written in the hope of highlighting the importance of maintaining mental wellbeing amongst the medical team, supporting help-seeking behaviour and changing attitudes toward mental health disorders amongst clinicians.

## Introduction

Lack of mindfulness, burnout, anxiety, depression, shift work, irregular work hours, as well as high-pressure environments are all recognized as adversely affecting clinicians’ mental wellbeing. Clinicians are vulnerable to mental illness; however, there are limited recommendations on how to recognize and support healthcare providers who may be affected by these issues. The World Health Organization’s definition of mental health is not simply the absence of mental illness, but is rather a “a state of well-being in which the individual realizes his or her own abilities, can cope with the normal stresses of life, can work productively and fruitfully, and is able to make a contribution to his or her community”. This definition lends itself to potential misunderstandings when it identifies positive feelings and positive functioning as key factors for mental health. Therefore, the European Psychiatric Association proposed a more inclusive definition reflecting human life experience and defines mental health as: “a dynamic state of internal equilibrium which enables individuals to use their abilities in harmony with universal values of society. Basic cognitive and social skills, ability to recognize, express and modulate one’s own emotions, as well as empathize with others, flexibility and ability to cope with adverse life events and function in social roles and harmonious relationship between body and mind represent important components of mental health which contribute, to varying degrees, to the state of internal equilibrium”.

This special contribution has been written on behalf of the editors of the International Urogynecology Journal in the loving memory of our colleague, friend and board member Dr. Nikolaus Veit-Rubin who sadly passed away on 20 January 2021. Our aim is to shine light on the mental health conditions common among the clinician population and identify perceived barriers to seeking help. In addition, we want to increase awareness of mental health issues among healthcare professionals and the consequences of not recognizing and seeking appropriate help. We hope that this will help our readership’s understanding of the importance of maintaining mental health among clinicians, and to support help seeking behaviour and change attitudes toward our colleagues struggling with psychological difficulties.

## Anxiety and depression

Almost half of all medical students suffer from anxiety symptoms at some point during their training. However, once their training is completed, practicing clinicians have similar rates of depression to the general population, but there is a higher prevalence of emotional exhaustion, depersonalization and burnout. In addition, the rates of suicidal ideation and suicide attempts are higher and more lethal than in the general population, suggesting that further research might be needed in this area.

Medical training involves high levels of internal drive, dedication and self-discipline, which is often also associated with neuroticism, perfectionism and self-criticism. Although these traits can be adaptive and helpful, they can also lead to heightened anxiety and depression, which is often the case in our profession where performance expectations are extremely high. These expectations can act a barrier to seeking appropriate help, where the self-critical clinician struggles to live up to the often unrealistically high standards of performance and misinterprets symptoms of anxiety and depression as a sign of weakness or incompetence. In response, the self-critical clinician may react with guilt and shame, withdrawing into worsening anxiety and depression rather than seeking social support. This is particularly important to consider when the clinician may be involved in medical errors, which are further associated with feelings of guilt, self-criticism, depression, fear and diminished self-worth. Problematically, anxiety and depression in clinicians are associated with compassion fatigue, impaired delivery of care and increased frequency of medical errors. This leads to a self-perpetuating cycle of depression and worsening clinical outcomes, bringing about further isolation and depression.

Despite growing awareness of anxiety and depression among the medical profession, there remains a barrier to seeking treatment. Studies show that time limitations, lack of convenient access, perceived stigma of mental illness and its impact of medical licensure prevent appropriate management among the medical profession. This often leads to “informal” consultations, self-diagnosis and management.

The impact of mental health on the ability to practice medicine is a valid concern for many clinicians; however, while protecting the patients, we must also protect the clinician. Currently, in most settings, mental health issues must be declared to your employer, along with treatment plans and, on return to practice, safe-to-practice letters.

## Suicide

The global suicide estimate rates are 10.6 per 100, 000 population (both sexes), with slightly higher rates in men at 13.5 per 100,000 compared with 7.7 per 100, 000 in women. Surprisingly, the suicide rate among male clinicians is 1.41 times higher than female clinicians and 2.27 times higher than in the general population.

Although clinicians have always been identified as an “at-risk” group, the subject remains taboo, often because of persistent notions of perfection and discomfort with the issue among clinicians. Research on suicidal behaviours in clinicians and medical students is limited, with small sample sizes, limited responses to questionnaires, fears of negative consequences related to disclosures and breaches of confidentiality.

It is believed that the prevalence of suicide ideation among medical students has an overall pooled crude prevalence of 11.1%. Interestingly, this risk seems to increase with progression through medical training, with rates rising from of 1.4% among 1st and 2nd year students to 7.9% during their 3rd and 4th years.

The prevalence of suicide ideation, usually due to severe depression, is believed to be the highest among medical students and thankfully seems to decrease incrementally with the passing of each stage in their early career. In fact, suicidal ideation has been reported in 9.4% of medical students, 8.1% of residents and fellows and 6.6% of early career clinicians with no statistically significant difference by year of residency or gender [1].

Risk factors for suicidal ideation among clinicians are being female, living alone, having symptoms of depression, low subjective well-being, poor self-reported health and high psychosocial work stress [2].

Among the various specialities, vascular surgeons have the highest and neurosurgeons the lowest rates of suicide. In a study of 7,905 surgeons, Shanafelt et al. found that 501 (6.3%) reported some suicidal ideation during the previous 12 months. Furthermore, they found that among individuals 45 years and older, suicidal ideation was 1.5–3.0 times more common among surgeons than in the general population [3]. More notably was that only 26% of surgeons with recent thoughts of suicide sought psychiatric or psychological help, and 60% were reluctant to pursue treatment owing to concerns that this would adversely affect their medical licence. A similar finding was noted among academic clinicians in France, Sweden and Italy. In this study the vast majority (106) of the 155 clinicians who reported recent thoughts of suicide had not sought mental health care, especially male academic clinicians. Using linear regression analysis, four independent correlates were found to be related to suicidal ideation: the burnout dimension of emotional exhaustion, lasting health problems, number of physical symptoms and lower body mass index. Interestingly, neither age nor gender was significantly associated with suicidal ideation [4].

Sadly, one of the most significant differences between the general population and the clinician is that clinicians are less likely to have a history of attempted suicide, which can help to identify the “at risk” individual. This is because clinicians are more successful with their first suicide attempt. Suicidal clinicians are profoundly serious about their intent and often obsessively research and rehearse a method that will ensure a lethal result. They use a means that will not only relieve their suffering, but that is foolproof. Drug overdose was the preferred method of suicie, and clinicians made more successful attempts than nurses [5].

## Burnout

Although the definition of burnout has been greatly debated, and is evolving, it is emotional exhaustion, depersonalization and a reduced feeling of personal accomplishment due to the chronic stress of helping others who are struggling. It is different to other common mental health conditions in that it is solely related to interpersonal stress in the workplace and does not include suicide ideation or feelings of hopelessness.

The prevalence of burnout is highest among medical students (45-71%) and it is believed to be related to high levels of stress, long hours of study, examinations, debt, relationship difficulties and mistreatment.

Among resident trainees, the rate of burnout increased from 17 to 46% from the first year to second year and then stabilized at that level until the senior year [6]. Compared with the general population, clinicians are 9% more likely to suffer from burnout, with repeated studies showing a 10% increase in rates of burnout reported by clinicians over the 3-year intervals [7].

Clinicians do not typically seek help, again, because of the fear of stigma, discrimination and concerns about confidentiality. This can lead to maladaptive coping strategies that include psychiatric disorders.

Anxiety, disorganization, personality traits such as pessimism and perfectionism, lack of coping skills, lack of autonomy, time pressure, poor relationships with colleagues, lack of work control, lack of work-life balance, lack of collegial support, type of practice setting, lack of financial rewards, number of hours worked per week and number of nights on call, fear of lawsuits, patient suffering, poor leadership, poor support staff, lack of sleep and death of patients were all predictive of burnout [8]. Most clinicians can identify with experiencing some or all these conditions at some point during their career.

## The impact of COVID-19 on clinician mental health

The pandemic has placed healthcare services under excess pressure, making achieving a work–life balance even more stressful than normal. The clinical workload has increased significantly with added pressures of redeployment, virtual clinics [9], sicker patients, stretching of limited resources including staff numbers and workspace and loss of normal teams [10, 11]. In addition, the stresses of the pandemic add an additional dimension of uncertainty owing to the fears of lack of personal protection equipment, new redeployment duties and of transmission of infection to themselves and their families. Under certain circumstances, social distancing deprives the clinicians of a crucial buffer against mental health difficulties, particularly for many clinicians who may be living apart from their families because of the risk of transmission or individuals living alone who lose their social network of friends and family owing to constant quarantining following exposures.

A recent systematic review by Kisely et al. showed that healthcare professionals during pandemics suffered higher levels of psychological distress [12]. Risk factors included younger age, being parents, having infected family members and prolonged time in quarantine.

During COVID-19, a survey among obstetrician/gynaecologists reported significantly higher rates of both major depressive disorder ( *p* = 0.023) and generalized anxiety disorder (*p* = 0.044) compared with the UK-wide estimates. Sub-group analysis showed that anxiety was more common among female doctors than among males (*p* = 0.047) [13].

The impact of school closures and lack of childcare support puts many clinicians, particularly women, under greater pressure to create a balance between work and life. Although this has always been a struggle, in the current climate of widespread home-schooling, the frontline clinician who does not have the luxury of working from home will need to use up annual leave with the guilt of leaving the team short-handed.

The social initiatives such as “Clap for Heroes”, healthcare discounts and free food in hospitals are welcome and provide some feelings of appreciation, but can feel superficial in comparison with what is truly needed. In the current environment, clinicians have little or no control over their clinical activities, which are usually decided by higher management, with the clinician being left to answer to the patient regarding cancellations or a return to normal clinical services.

## Prevention and coping strategies

Healthcare executives and managers, indeed all of us, need to be aware that clinicians are at a higher risk of psychological stress and suicidal ideation, particularly during a pandemic. The literature shows that healthcare professionals place a high value on the provision of training and equipment during pandemics; however, effective leadership and managerial support for clinicians and their families are highly protective against negative psychological outcomes.

Unfortunately, there are limited studies looking at mental health among urogynaecologists, with most of the literature focusing more generally on the specialty of obstetrics and gynaecology.

Urogynaecologists are part of the same medical family, which includes all specialties in the medical field. In other words, we are all doctors, we all have similar human feelings, we all have family and sometimes psychosocial problems to face. Therefore, mental health disorders are common issues that we need to learn to recognize and manage in the best way possible.

It is important to remember that doctors are less likely to seek help; hence, increasing awareness of mental health issues among healthcare professionals is imperative. Workplace interventions that reduce mental health stigma and promote support for colleagues with psychological difficulties might improve help-seeking behaviour and attitudes to mental health. Awareness, support from family, colleagues and friends, as well as mindfulness practice, are viable techniques for helping groups or individual clinicians to manage stress.

## Appendix

Below are some of the very helpful resources that our readership may find helpful to better understand the importance of mental health among clinicians:

IUGA wellness resources. Available from URL https://www.iuga.org/resources/wellness-resources

RANZCOG offers trainees confidential counselling sessions with accredited therapists over the telephone or videoconferencing through Converge, its Member Support Program. RANZCOG. Member Support and Wellbeing, 2020. RANZCOG. Available from URL https://ranzcog.edu.au/members/member-support-and-wellbeing

Self-care has always been important but even more so at this time of social distancing. The RCOG recommends https://www.liverpool.ac.uk/project-ares/preventingptsd, which we found to have several useful resources on how to detect early signs of emotional distress in oneself and in peers, when to seek help, and how to cope.

American Foundation for Suicide Prevention. https://afsp.org/get-help

NHS Practitioner Health Programme (PHP) (www.php.nhs.uk).

Teaandsympathy: Facebook, Tea & Empathy, a national, informal, peer-to-peer support network aiming to foster a compassionate and supportive atmosphere throughout the NHS.

The Centre for Mind-Body Medicine, https://cmbm.org/

https://www.datocms-assets.com/12810/1578318836-after-a-suicide-a-toolkit-for-physician-residency-fellowship-program.pdf

Project 2025: Raising Awareness for Suicide Treatment. Cams-care. Two continuing education credits available via online on-demand webinar. https://cams-care.com/resources/events/suicide-prevention-healthcare-settings/
